# A novel IL-1RA-PEP fusion protein alleviates blood-brain barrier disruption after ischemia-reperfusion in male rats

**DOI:** 10.1186/s12974-018-1058-z

**Published:** 2018-01-15

**Authors:** Dong-Dong Zhang, Chen Jin, Ya-Tao Zhang, Xiang-Dong Gan, Min-Ji Zou, Yuan-Yuan Wang, Wen-Liang Fu, Tao Xu, Wei-Wei Xing, Wen-Ron Xia, Dong-Gang Xu

**Affiliations:** 1 Beijing Institute of Basic Medical Sciences, 27 Taiping Road, Beijing, 100850 People’s Republic of China; 2 Anhui Medical University, 81 Meishan Road, Hefei, 230032 People’s Republic of China; 3 Laboratory of Genome Engineering, Beijing Institute of Basic Medical Sciences, 27 Taiping Road, Beijing, 100850 People’s Republic of China

**Keywords:** Ischemia-reperfusion injury, Blood-brain barrier, IL-1RA-PEP fusion protein, Middle cerebral artery occlusion, Angiogenesis

## Abstract

**Background:**

Current options to treat clinical relapse in inflammatory central nervous system (CNS) conditions such as cerebral ischemia-reperfusion injury are limited, and agents that are more effective are required. Disruption of the blood-brain barrier is an early feature of lesion formation that correlates with clinical exacerbation and facilitates the entry of inflammatory medium and inflammatory cells. Interleukin-1 receptor antagonist (IL-1RA) is a naturally occurring anti-inflammatory antagonist of the interleukin-1 (IL-1) family. The broad-spectrum anti-inflammatory effects of IL-1RA have been investigated against various forms of neuroinflammation. However, the effect of IL-1RA on blood-brain barrier disruption following ischemia-reperfusion has not been reported.

**Methods:**

In this study, we investigated the effects of IL-1RA and a novel protein (IL-1RA-PEP) that was fused to IL-1RA with a cell penetrating peptide, on blood-brain barrier integrity, in male rats subjected to transient middle cerebral artery occlusion.

**Results:**

After intravenous administration, IL-1RA-PEP (50 mg/kg) penetrated cerebral tissues more effectively than IL-1RA. Moreover, it preserved blood-brain barrier integrity, attenuated changes in expression and localization of tight junction proteins and matrix metalloproteinases, and enhanced angiogenesis in ischemic brain tissue. Further study suggested that the effects of IL-1RA-PEP on preserving blood-brain barrier integrity might be closely correlated with the p65/NF-κB pathway, as evidenced by the effects of the inhibitor JSH-23.

**Conclusions:**

Collectively, our results demonstrated that IL-1RA-PEP could effectively penetrate the brain of rats with middle cerebral artery occlusion and ameliorate blood-brain barrier disruption. This finding might represent its novel therapeutic potential in the treatment of the cerebral ischemia-reperfusion injury.

## Background

The blood-brain barrier (BBB) acts as a selective interface that insulates the brain parenchyma from the blood circulation. It is comprised of endothelial cells, pericytes, astrocytes, neurons, and the extracellular matrix [1]. Increased BBB permeability is an early and prominent feature of neuropathological diseases, including ischemia-reperfusion injury, stroke, and subarachnoid hemorrhage (SAH) [2–4]. Ischemia-reperfusion injury can lead to damage of the BBB that is correlated with the processes of oxidative stress, neuroinflammation, apoptosis, excitotoxicity, and intracellular calcium overload. Disruption of the BBB results in edema, metabolic imbalances, and ingress of inflammatory factors, and facilitates infiltration of T and B lymphocytes, macrophages, and neutrophils. Therefore, protection of the BBB is one of the important targets in the treatment of ischemia-reperfusion injury [5–7].

A considerable number of studies in various cerebral diseases indicate that permeability of the BBB is closely associated with tight junction (TJ) proteins, matrix metalloproteinases (MMPs), and microvascular endothelial cells (MVECs) [8–10]. The TJ proteins (ZO-1 and occludin) play key roles in junction formation at the BBB [11]. The MMPs (MMP-2 and MMP-9) regulate the properties of the BBB [12]. The MVECs use complex tight junctions and restrict the permeability of the BBB [13]. Thus, the establishment, maintenance, and repair of TJs, MMPs, and MVECs are all linked to BBB disruption.

As a member of the IL-1 family of cytokines, IL-1RA can competitively bind with the interleukin-1 receptor (IL-1R), to antagonize inflammatory effects of the interleukin-1α (IL-1α) and interleukin-1β (IL-1β) [14]. In studies of various cerebral conditions, including stroke, SAH, and brain trauma [15–18], IL-1RA has been observed to have broad-spectrum anti-neuroinflammatory effects. However, to our knowledge, the effects of IL-1RA on BBB disruption, following ischemia-reperfusion injury, have not been reported. In addition, the effective penetration of IL-1RA (intravenously administered) in cerebral tissue is not very high because of its relatively high molecular weight (17 kDa).

Cell-penetrating peptides (CPPs) have the ability to act as transmembrane vectors that can deliver various biomolecules and drugs across diverse biomembranes, including the BBB, gastroenteric mucosa, and dermis, to facilitate normal biological functions [19–21]. As a short amphipathic peptide carrier and a CPP, PEP-1 is extensively involved in the transport of biomolecules into various tissues and organs. Because of its stability in physiological buffers and lack of toxicity, PEP-1 possesses exceptional advantages for protein delivery in vivo [22]. Thus, in this study, we constructed a novel bi-functional protein, IL-1RA-PEP, by fusing IL-1RA with PEP-1. We used it in the middle cerebral artery occlusion (MCAO) rat model in vivo to test whether IL-1RA-PEP has enhanced brain penetration in comparison to IL-1RA. We also sought to investigate whether this novel protein has improved protective effects on BBB disruption. In addition, we further elucidated the possible pharmacological mechanisms underlying its action. We predicted that PEP-1 might enhance the effects of IL-1RA to effectively alleviate BBB disruption via improvement of the permeation efficiency of IL-1RA to brain tissue.

## Methods

### Animals

Male Sprague-Dawley rats (250–290 g) were purchased from Vital River Co., Ltd. (Beijing, China). All rats were fed at the standard laboratory animal facility (25 °C, 12-h light/dark cycle) with ad libitum access to food and water for at least 2 weeks before the study.

### Animal surgery and drug administration

Male Sprague-Dawley rats were subjected to transient MCAO, as reported previously [23]. Briefly, rats were anesthetized with 4% isoflurane (NO_2_/O_2_, 70%/30%). A nylon filament, 2 cm in length and diameter (*φ*) of 0.26 mm, was inserted into the right internal carotid artery (ICA) to occlude the origin of the right middle cerebral artery. After 2 h of MCAO, reperfusion was accomplished by withdrawing the filament. The body temperature of rats was maintained at 37.0 ± 0.5 °C throughout the procedure using a temperature control system. All rats were operated by the same surgeon in similar conditions to reduce variability.

The rats were randomly assigned to four groups. In the IL-1RA-PEP group, IL-1RA-PEP (a recombinant protein comprising fused IL-1RA and PEP-1, described in our previous study [24]) was diluted with saline and administered intravenously (50 mg/kg) 2 h after transient MCAO followed by reperfusion. In the vehicle group, saline was administered in the same manner. In the IL-1RA group, IL-1RA (50 mg/kg, a recombinant human protein prepared in our previous study [24]) was administered intravenously 2 h after transient MCAO followed by reperfusion. In the control group, rats underwent the same operation, without occlusion of the middle cerebral artery.

### Evaluation of Evans blue dye extravasation

According to previously described methods [25], Evans blue (EB) dye (Sigma-Aldrich, St. Louis, MO, USA, 4% in saline, 3 mL/kg) was injected into the tail vein 22 h after transient MCAO. Two hours after injection of the EB dye, rats were anesthetized and transcardially perfused with 250–300 mL saline at room temperature. The brains of the rats were then removed, weighed, cut up, and soaked in formamide (1 mL/100 mg) at 55 °C for 24 h. Supernatants were obtained by centrifugation at 10,000×*g* for 10 min at 4 °C. The EB content in the tissue samples was measured as the level of fluorescence at 620 nm. It was then quantified using a linear regression standard curve derived from eight concentrations (0–10^4^ ng/mL) of the dye and expressed as μg/g of tissue.

### Examination of BBB ultrastructure

Transmission electron microscopy (TEM) was used to determine BBB ultrastructure [26]. Twenty-four hours after ischemia-reperfusion injury, rats were anesthetized and perfused transcardially with 2% glutaraldehyde in 0.1 mol/L phosphate buffer. Approximately 1 mm^3^ of the ischemic penumbra of the cortex was taken and fixed in freshly prepared 3% glutaraldehyde overnight at 4 °C and post-fixed in 1% osmium tetroxide for 2 h. The specimens were then dehydrated through a graded series of ethanol and embedded in Epon 812. Ultrathin sections of the cortex were obtained and doubly stained with uranyl acetate and lead citrate. Sections were then examined using TEM (JEOL-1011, JEOL, Tokyo, Japan).

### Immunohistological detection of endogenous IgG leakage

Leakage of endogenous IgG was also used to assess BBB disruption, according to previously described methods, with minor modifications [27]. Rats were anesthetized with isoflurane and then perfused transcardially with 0.9% saline, followed by 4% paraformaldehyde (PFA) in 0.1 mol/L phosphate buffer (PB, pH = 7.4). The brains of the rats were removed after 20 min perfusion fixation and then immersed in 4% paraformaldehyde overnight at 4 °C. The brains were then cryoprotected with 30% sucrose in 0.1 mol/L PB for 24 h and then sliced into 20-μm coronal sections in a cryostat. Coronal sections were stored at − 80 °C until further use.

Slides with the sections were completely air dried, treated with 0.01 mol/L sodium citrate buffer (pH = 6.0) for antigen retrieval, and washed three times with phosphate-buffered saline (PBS). After blocking with 10% normal goat serum (Vector Laboratories, Burlingame, CA, USA) at room temperature for 1 h, the sections were incubated overnight at 4 °C with Cy® 3 conjugated goat anti-rat IgG antibody (1:300, Molecular Probes, Eugene, OR, USA) under protection from light. After three rinses with PBS, 4′,6-diamidino-2-phenylindole (DAPI) (molecular probes) was applied to stain all the nuclei. The brain sections were mounted, coverslipped, and photographed under a Nikon A1 confocal on a Ti-E microscope (Nikon, Sola, Sweden).

### Intravenous injection and measurement of dextran tracer

For quantitation of BBB permeability, another set of rats subjected to ischemia-reperfusion injury was injected with fluorescein isothiocyanate-dextran (FITC-dextran) (40 kDa, 1 mg/kg body weight, Sigma) in the tail vein 1 h before sacrifice. In the same manner as described above, frozen serial coronal brain sections were prepared, stained with DAPI, and visualized directly under a fluorescent microscope. Images were captured, and distribution of the FITC-dextran tracer was calculated using ImageJ software.

### Immunohistochemical analysis of brain penetration

Immunohistochemical staining was also used to detect penetration of IL-1RA and IL-1RA-PEP in the brain. Coronal frozen sections (20 μm) were produced in the same manner as described above. The sections were washed with PBS. They were then blocked with 10% normal rabbit serum for 30 min and incubated overnight at 4 °C with goat anti-human IL-1RA antibody (1:500, R&D Systems, Inc., Minneapolis, USA) and rabbit anti-NeuN antibody to label neuronal cells (1:100, Chemicon, Hampshire, UK). Brain sections were washed and then incubated with appropriate fluorochrome-conjugated secondary antibodies (Alexa Fluor 488 or Cy® 3, Invitrogen, Carlsbad, CA, USA) for 1 h at room temperature. The sections were counterstained with DAPI. Images were photographed with laser scanning confocal fluorescence microscopy (Zeiss LSM780, Carl Zeiss, Jena, Germany).

### Enzyme-linked immunosorbent assay (ELISA) quantitation for brain distribution

In a separate study, a single intravenous dose of IL-1RA (50 mg/kg) or IL-1RA-PEP (50 mg/kg) was administered at the time of reperfusion after MCAO for 2 h. Four hours after intravenous administration, rats were anesthetized and perfused transcardially with ice-cold PBS at 15 mL/min for 3 min. Rats were then decapitated, and the whole brain was collected. The isolated brain sample was separated on ice into cerebral cortex and striatum. These samples were weighed, and homogenized with a glass tissue grinder, in PBS (10 times the volume of the samples) containing protease inhibitors. The homogenized samples were centrifuged at 4 °C and 13,200 × *g* for 15 min. The IL-1RA concentrations in the supernatant of the brain tissue homogenates were determined using the Quantikine® ELISA kit (Human IL-1ra/IL-1F3, R&D Systems lnc., Minneapolis, USA).

### Immunofluorescence staining

Brain coronal frozen sections (20 μm) were produced in the same manner as described above. The sections were blocked with 10% normal goat serum (Vector Laboratories, Burlingame, CA, USA) at room temperature for 1 h and then incubated with primary antibodies overnight at 4 °C. The primary antibodies used were as follows: rabbit anti-occludin (1:50, Proteintech Group Inc., Rosemont, USA), rabbit anti-ZO-1 (1:50, Proteintech Group Inc.), rabbit anti-claudin-5 (1:200, Affinity Biosciences, OH, USA), and mouse anti-vWF to label endothelial cells (1:50, Millipore, Temecula, CA, USA); or rabbit anti-MMP-2 (1:100, Abcam Inc., Cambridge, MA, USA), rabbit anti-MMP-9 (1:1000, Cell Signal Technology, Boston, USA), and mouse anti-GFAP to label astrocyte cells (1:100, Cell Signal Technology). Brain sections were rinsed with PBS and then incubated with Cy® 3 conjugated goat anti-rabbit IgG antibody (1:500, Invitrogen, Carlsbad, CA, USA) and Alexa Fluor 488 conjugated goat anti-mouse IgG antibody (1:300, Molecular Probes) for 1 h at room temperature. All nuclei were stained with DAPI (molecular probes). After being washed, the sections were observed under a laser scanning confocal microscope (FV10i, Olympus, Tokyo, Japan).

### Real-time quantitative PCR

Rats were deeply anesthetized and decapitated 24 h after ischemia-reperfusion. Their brains were immediately resected, and the ischemic cerebral cortices were separated and stored at − 80 °C until further use.

Total RNA was extracted from frozen ischemic cerebral cortices. The RNA was reverse-transcribed and amplified using reverse transcriptase (RT)-PCR (7900 Real-time PCR System, Life Technologies, USA). The primers of vascular endothelial growth factor (VEGF), angiopoietin-1 (Ang-1), and glyceraldehyde 3-phosphate dehydrogenase (GAPDH) were designed as follows: VEGF, forward primer: 5′-CAC CAA AGC CAG CAC ATA GG-3′, reverse: 5′-TTT AAC TCA AGC TGC CTC GC-3′; Ang-1, forward: 5′-TGA TGG ACT GGG AAG GGA AC-3′, reverse: 5′-CAC AGG CAT CAA ACC ACC AA-3′; GAPDH, forward: 5′-AAG ATG GTG AAG GTC GGT GT-3′, reverse: 5′-TGA CTG TGC CGT TGA ACT TG-3′. The ΔΔCt values from each group were analyzed, and mRNA expression levels were normalized to 2-ΔΔCt. Expression of the specific genes of interest was compared across groups.

### Western blotting

The isolated ischemic cerebral cortices were homogenized in ice-cold radioimmunoprecipitation assay (RIPA) lysis buffer (CWBiotech, Beijing, China), containing a protease inhibitor cocktail (Roche, Indianapolis, IN, USA) for the extraction of protein samples. Protein concentration was determined using the bicinchoninic acid (BCA) method. Equal amounts of proteins (100 μg) were separated on SDS-polyacrylamide gels at 100 V for 80–120 min and transferred to polyvinylidene difluoride (PVDF) membranes (Millipore Corporation, Billerica, MA, USA) at 250 mA for 100 min. The membranes were blocked with 5% nonfat dried milk in tris-buffered saline Tween-20 (TBST) for 1 h at room temperature and then incubated overnight at 4 °C with primary antibodies against occludin, ZO-1, MMP-2, MMP-9 (1:1000, Proteintech Group Inc.), claudin-5 (1:500, Affinity Biosciences), VEGF, Ang-1 (1:1000, Abcam Inc.), and GAPDH (1:1000, CWBiotech). The membranes were washed and then incubated with the respective horseradish peroxidase-conjugated secondary antibodies (1:2500, ZSGB-BIO, Beijing, China) for 1 h at 37 °C. Immunoreactive bands were visualized using a chemiluminescent detection system kit (Millipore Corporation, Billerica, MA, USA). Band intensities were measured with the ImageJ software (National Institute of Health [NIH], Bethesda, MD, USA).

After the NF-κB inhibitor, JSH-23 (50 mg/mL) (Selleck Chemicals, USA), was added, ZO-1, MMP-9, Ang-1, and VEGF were again detected by the methods described above.

### Statistical analysis

All data were analyzed with the statistical software, GraphPad Prism 5 (La Jolla, CA, USA) and presented as mean ± SEM. The parametric data of two groups were analyzed using the Student’s *t* test, and Welch’s correction was applied for data with unequal variances. All differences in other data between groups were assessed by one-way ANOVA analysis, followed by the Bonferroni test for multiple comparisons. Statistically significant, very significant and highly significant differences were determined by *P* < 0.05, *P* < 0.01, and *P <* 0.001, respectively.

## Results

### Effects of IL-1RA and IL-1RA-PEP on BBB disruption in rats following transient MCAO

**Fig. 1:**
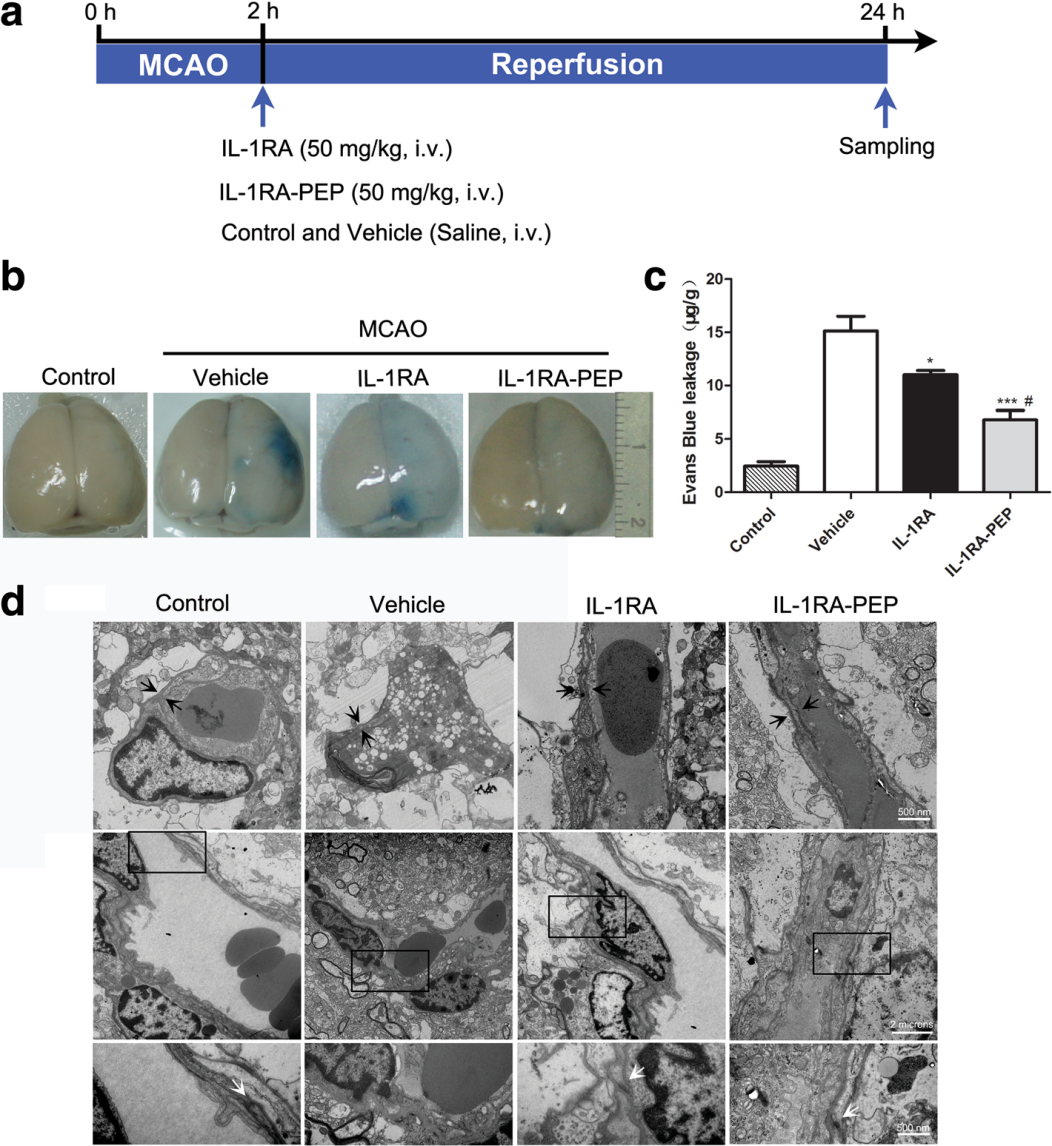
Effects of IL-1RA and IL-1RA-PEP on transient MCAO-induced Evans blue extravasation and BBB ultrastructure. **a** Experimental protocol. **b**, **c** Extravasation of Evans blue dye in brain tissues of the control, vehicle, IL-1RA, and IL-1RA-PEP groups was evaluated quantitatively. Data are shown as mean ± SEM; *P < 0.05, **P < 0.01, ***P < 0.001 compared to the vehicle group, #*P* < 0.05 compared to the IL-1RA group, *n* = 6 per group, based on one-way ANOVA with Bonferroni correction. **d** Ultrastructure changes in the BBB. The basement membranes (marked between black arrows in top panels) were observed in different groups. The black frames in middle panels are areas selected for amplification in the following bottom panels, to highlight tight junctions (pointed by white arrows). Scale bar, 500 nm and 2 μm for the inserts. *MCAO* middle cerebral artery occlusion, *BBB* blood-brain barrier

The experimental protocol is presented in Fig. 1a. Extravasation of Evans blue, the BBB ultrastructure, leakage of endogenous IgG, and permeability of FITC-dextran tracer in the brain were all used to evaluate BBB breakdown 24 h after ischemia-reperfusion.

Evans blue extravasation in the vehicle group was notably exacerbated, in comparison to the control group, whereas extravasation of Evans blue in IL-1RA-PEP (50 mg/kg)-treated rats was significantly alleviated, in comparison to the vehicle group (6.79 ± 0.88 versus 15.12 ± 1.39 μg/g, *P* < 0.001). Extravasation was also significantly reduced in the positive IL-1RA (50 mg/kg) group (11.00 ± 0.41 μg/g), in comparison to the vehicle group. Moreover, extravasation in the IL-1RA-PEP group was significantly lower than that in the IL-1RA group (*P* < 0.05) (Fig. 1b, c).

Ultrastructural changes of the BBB were examined in the different groups (Fig. 1d). The complete basement membrane and tight junctions were observed in the control group. After the ischemia-reperfusion injury, the basement membrane was disrupted and tight junctions were damaged in the vehicle group. However, in the IL-1RA-treated and IL-1RA-PEP-treated groups, the basement membrane was preserved and the tight junctions were slowly restored.

**Fig. 2:**
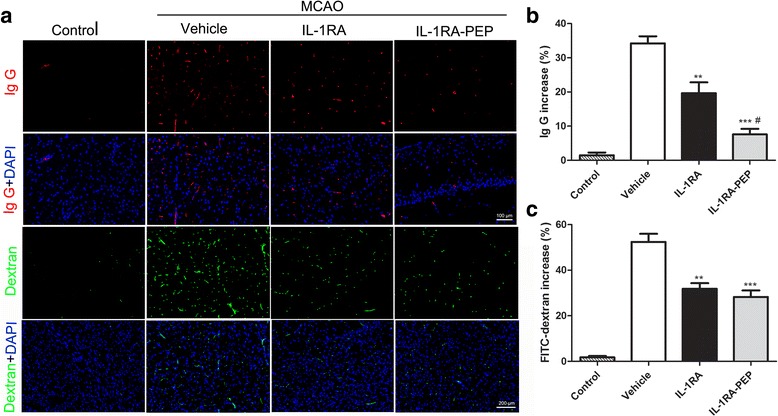
Effects of IL-1RA and IL-1RA-PEP on transient MCAO-induced endogenous IgG leakage and permeability of FITC-dextran. **a** Extravasation of endogenous IgG, and intravenous injection and measurement of FITC-dextran tracer were evaluated in the different groups. Increases (%) in IgG (**b**) and FITC-dextran (**c**) were represented by the proportion of the areas of positive regions in the cerebral cortex of the ipsilateral hemisphere. Scale bar, 100 and 200 μm for the inserts. Data are shown as mean ± SEM; **P < 0.01, ***P < 0.001 compared to the vehicle group, #*P* < 0.05 compared to the IL-1RA group, *n* = 6 per group, based on one-way ANOVA with Bonferroni correction. *MCAO* middle cerebral artery occlusion, *FITC-dextran* fluorescein isothiocyanate–dextran

In terms of endogenous IgG leakage, 24 h after MCAO, leakage in the vehicle group was more obvious than that in the control group. However, the leakage of endogenous IgG in the right cerebral hemisphere of IL-1RA-PEP-treated rats was very significantly brought down, in comparison to that in the vehicle rats (7.58% ± 1.62 versus 34.21% ± 2.08%, *P* < 0.001). Leakage (19.65% ± 3.16%) was also significantly reduced in the IL-1RA-treated group in comparison to the vehicle group. Furthermore, leakage in IL-1RA-PEP-treated rats was significantly lower than that in IL-1RA-treated rats (*P* < 0.05) (Fig. 2a, b).

The FITC-dextran permeability assay was performed to examine and quantify the changes in BBB integrity 24 h after ischemia-reperfusion injury. As shown in Fig. 2a, c, in comparison to control rats, cerebrovascular permeability to FITC-dextran in vehicle rats was significantly aggravated. The permeability of FITC-dextran was attenuated by post-ischemic treatment with IL-1RA-PEP (50 mg/kg), in comparison to the vehicle group (28.27% ± 2.84 versus 52.40% ± 3.57%, *P* < 0.001). Furthermore, in comparison to the vehicle group, permeability was significantly ameliorated by treatment with IL-1RA (50 mg/kg) (31.87% ± 2.45%) (*P* < 0.01).

### Brain penetration effects and levels of IL-1RA and IL-1RA-PEP

**Fig. 3:**
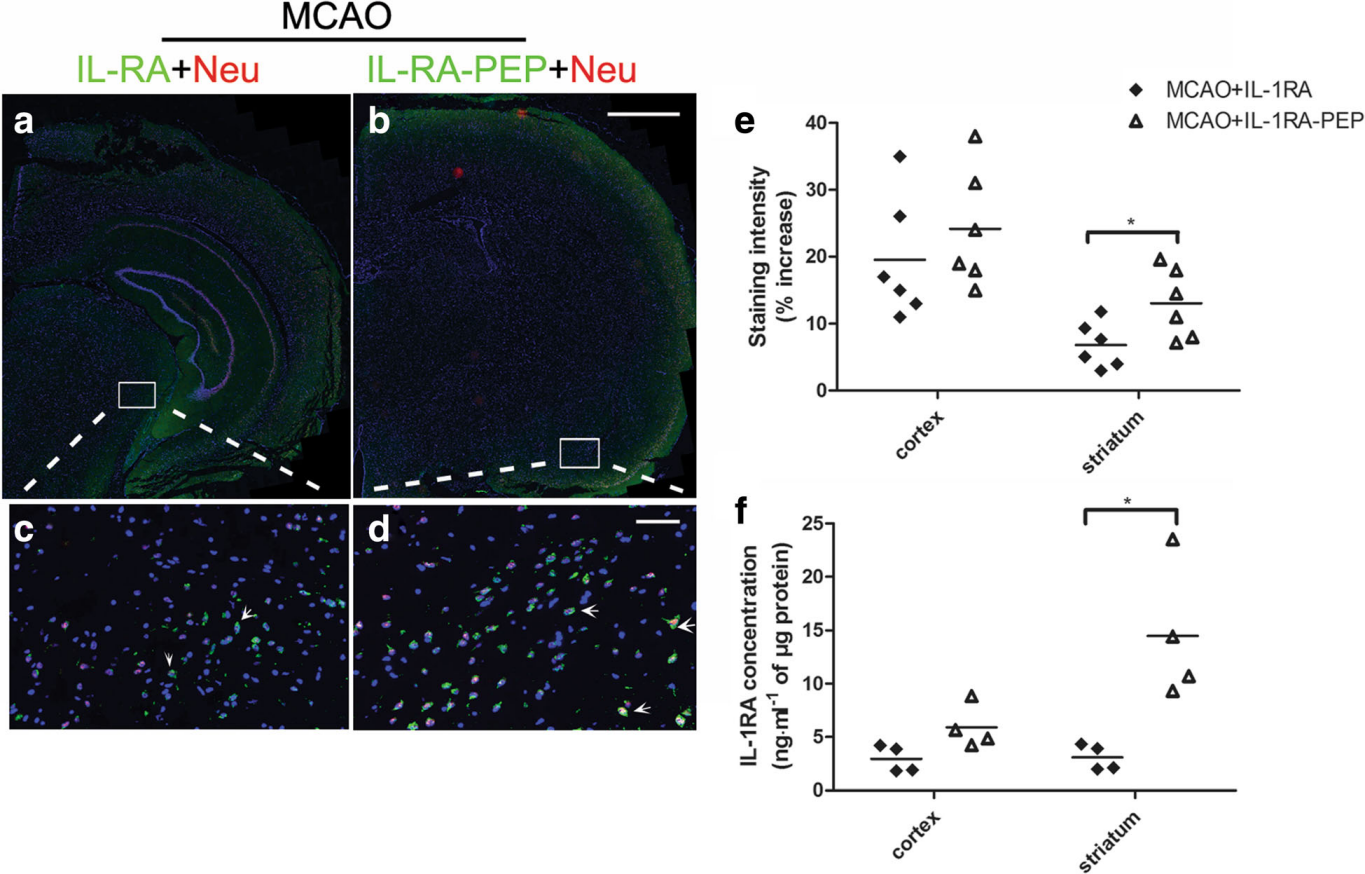
Brain penetration effects and levels of IL-1RA and IL-1RA-PEP. **a**, **b** At 24 h after ischemia-reperfusion, the permeation effect on brain tissue was assessed by immunohistochemical staining. The rats with middle cerebral artery occlusion (MCAO) in separate groups were injected intravenously with IL-1RA or IL-1RA-PEP. **c**, **d** High magnification of double immunofluorescence images show co-localization of IL-1RA with NeuN (arrows) and IL-1RA-PEP with NeuN (arrows). Scale bar, 5.0 mm and 50 μm for the inserts. **e** The permeation effect was expressed as a percentage increase in staining intensity in the cerebral cortex and striatum of the contralateral hemisphere. **f** The concentration of IL-1RA in the ipsilateral ischemic brain hemisphere of rats subjected to MCAO, 4 h after a single intravenous injection of IL-1RA (50 mg/kg) or IL-1RA-PEP (50 mg/kg), was measured by ELISA. Panels show IL-1RA concentration in the cerebral cortex and striatum area, respectively. Data are shown as mean ± SEM; *P < 0.05 in comparison with the rat intravenously injected with IL-1RA, *n* = 4–6 per group, based on the Student’s *t* test with Welch’s correction

The permeation effects of IL-1RA and IL-1RA-PEP on the brain of rats were analyzed by immunohistochemical technology. In rats subjected to MCAO that had been intravenously administered IL-1RA (50 mg/kg) or IL-1RA-PEP (50 mg/kg), a low concentration of IL-1RA (Fig. 3a) and a high concentration of IL-1RA-PEP were detected in the brain (Fig. 3b), respectively. Moreover, the magnification of double immunofluorescence staining indicated that IL-1RA only accumulated on the cell surface of neurons (Fig. 3c), but IL-1RA-PEP was able to both bind to IL-1R and penetrate neurons (Fig. 3d). In comparisons of the positive staining regions between the cerebral cortex and striatum, the staining intensity of IL-1RA-PEP that reached the brain was significantly higher than that of IL-1RA (in the striatum of MCAO rats, 13.05% ± 2.11% versus 6.82% ± 1.38%, *P* < 0.05) (Fig. 3e).

The permeation of IL-1RA and IL-1RA-PEP into brain tissues was analyzed by ELISA, 4 h after intravenous administration in rats subjected to MCAO. In comparisons of IL-1RA concentration per microgram of protein in the ischemic brain hemisphere, the concentration of IL-1RA-PEP that reached brain tissues (the ipsilateral cerebral cortex and striatum area) was significantly higher than that of IL-1RA (in the striatum of rats subjected to MCAO, 14.50 ± 3.20 versus 3.09 ± 0.60 ng mL^−1^, *P* < 0.05) (Fig. 3f).

These results show that IL-1RA-PEP possessed an enhanced capacity to penetrate brain tissue in comparison to IL-1RA, under conditions of ischemia-reperfusion injury.

### Effects of IL-1RA and IL-1RA-PEP on the transient MCAO-induced changes in TJ proteins

**Fig. 4:**
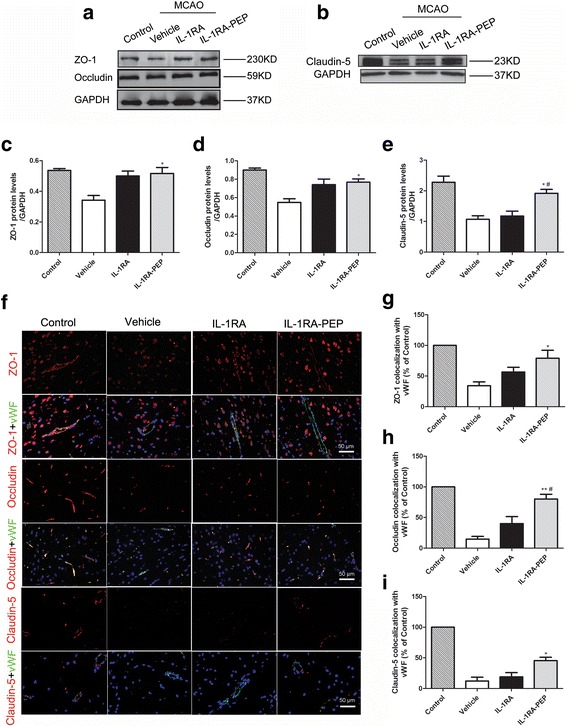
Effects of IL-1RA and IL-1RA-PEP on transient MCAO-induced changes in tight junction protein expression and localization. **a–e** Protein expression levels of ZO-1, occludin, and claudin-5 in cerebral tissue of the control, vehicle, IL-1RA, and IL-1RA-PEP groups were evaluated. **f–i** Localization of ZO-1, occludin, and claudin-5 in the cerebral tissue of different groups was also analyzed. Scale bar = 50 μm. Data are shown as mean ± SEM; *P < 0.05, **P < 0.01 compared to the vehicle group, #*P* < 0.05 compared to the IL-1RA group, *n* = 4 per group, based on one-way ANOVA with Bonferroni correction. *MCAO* middle cerebral artery occlusion

The TJ proteins, ZO-1, occludin, and claudin-5 are crucial proteins that are involved in the process of BBB breakdown following transient MCAO in rats [28]. Given this finding, the expression and localization of the TJ proteins ZO-1, occludin, and claudin-5 in brain tissue were investigated. In comparison to the control group, 24 h after ischemia-reperfusion, levels of ZO-1, occludin, and claudin-5 were significantly decreased in the vehicle group. However, in comparison to the vehicle group, the IL-1RA-PEP group showed a marked increase in these protein levels (ZO-1, 0.52 ± 0.04 versus 0.34 ± 0.03, *P* < 0.05; occludin, 0.77 ± 0.03 versus 0.55 ± 0.04, *P* < 0.05; claudin-5, 1.92 ± 0.13 versus 1.07 ± 0.12, *P* < 0.05) (Fig. 4a–e). Moreover, IL-1RA-PEP significantly enhanced the reconstitution of ZO-1, occludin, and claudin-5 proteins in cerebral tissues, following transient MCAO (ZO-1, 79.13% ± 12.98% versus 34.26% ± 6.19%, *P* < 0.05; occludin, 80.24% ± 7.72% versus 14.72% ± 4.44%, *P* < 0.01; claudin-5, 45.59% ± 5.41% versus 12.20% ± 6.33%, *P* < 0.05) (Fig. 4f–i). In addition, rats administered IL-1RA showed little improvement in the expression levels and distribution of these proteins, in comparison to rats in the vehicle group. Nevertheless, the expression of claudin-5 (1.18 ± 0.16) and redistribution of occludin (40.02% ± 11.51%) in rats administered IL-1RA were notably lower than those in rats administered IL-1RA-PEP (*P* < 0.05). The data indicate that MCAO-induced changes in characteristics of the TJ proteins were significantly restored by IL-1RA-PEP.

### Effects of IL-1RA and IL-1RA-PEP on the transient MCAO-induced changes in MMPs

**Fig. 5:**
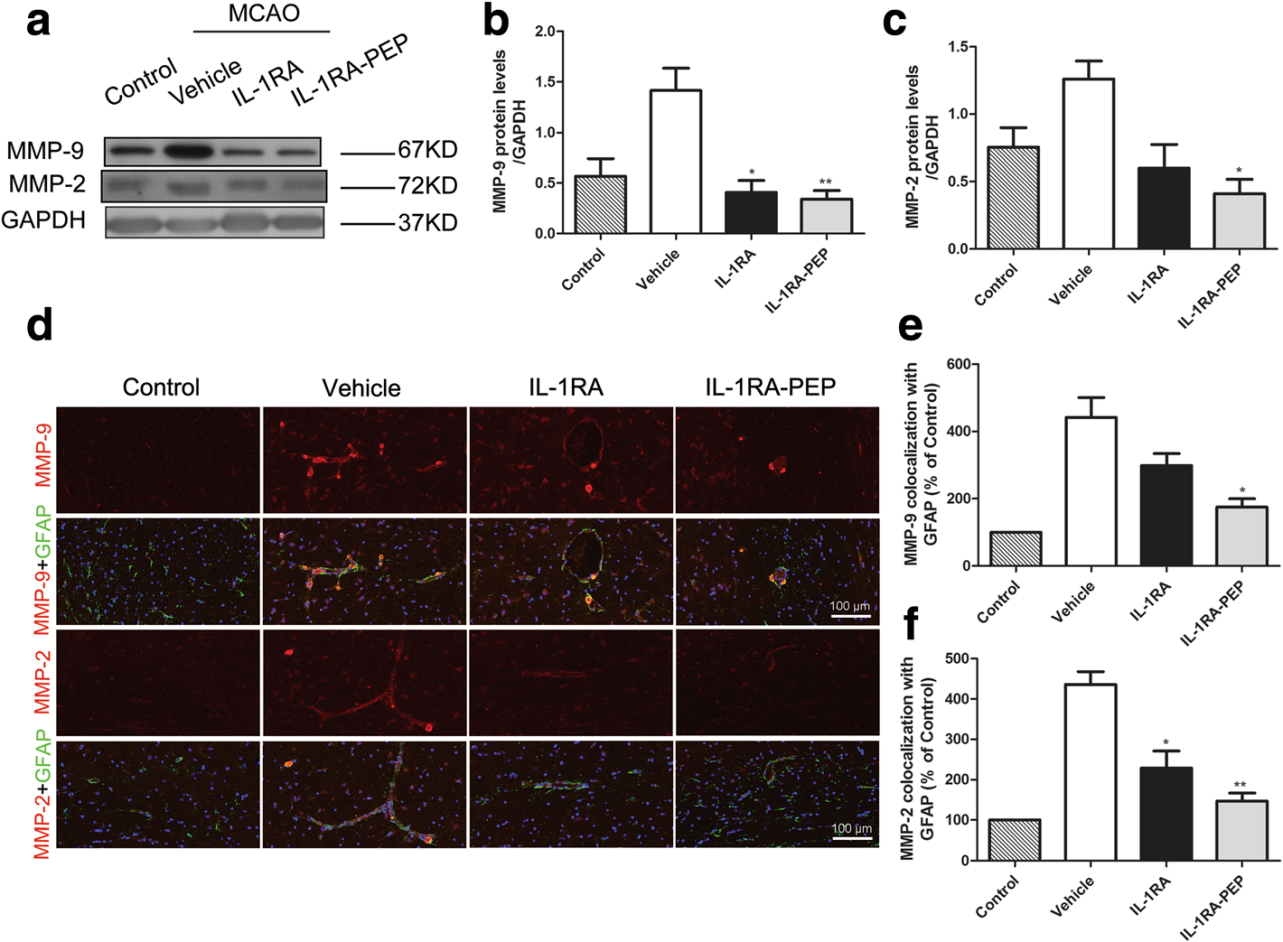
Effects of IL-1RA and IL-1RA-PEP on transient MCAO-induced changes in MMP protein expression and localization. **a–c** Protein expression levels of MMP-9 and MMP-2 in cerebral tissue of the control, vehicle, IL-1RA, and IL-1RA-PEP groups were evaluated. **d–f** Localization of MMP-9 and MMP-2 in the cerebral tissue of different groups was also analyzed. Scale bar = 100 μm for the inserts. Data are shown as mean ± SEM; *P < 0.05, **P < 0.01, ***P < 0.001 compared to the vehicle group, #*P* < 0.05 compared to the IL-1RA group, *n* = 4 per group, based on one-way ANOVA with Bonferroni correction. *MCAO* middle cerebral artery occlusion

Studies have shown that the expression and localization of MMPs strongly influence the integrity of the BBB [29]. In view of this, the effects of IL-1RA and IL-1RA-PEP on the expression and localization of MMPs in the brain, following transient MCAO were investigated. The levels of MMP-9 and MMP-2 in cerebral tissues of the vehicle group were markedly increased in comparison to the control group. Changes in these protein levels were attenuated by post-ischemic treatment with IL-1RA-PEP (MMP-9, 0.34 ± 0.09 versus 1.41 ± 0.22, *P* < 0.01; MMP-2, 0.41 ± 0.11 versus 1.26 ± 0.13, *P* < 0.05) (Fig. 5a–c). Additionally, transient MCAO-induced redistribution of MMP-9 and MMP-2 proteins in cerebral tissues of the IL-1RA-PEP-treated group was significantly suppressed in comparison to the vehicle group (MMP-9, 175.0% ± 24.7% versus 441.7% ± 59.4%, *P* < 0.05; MMP-2, 146.7% ± 20.33% versus 436.0% ± 31.7%, *P* < 0.01) (Fig. 5d–f). These results show that MCAO-induced transient changes in MMP characteristics were alleviated by IL-1RA-PEP, which might contribute to the BBB protection of IL-1RA-PEP.

### Effects of IL-1RA and IL-1RA-PEP on transient MCAO-induced changes in angiogenic factors

**Fig. 6:**
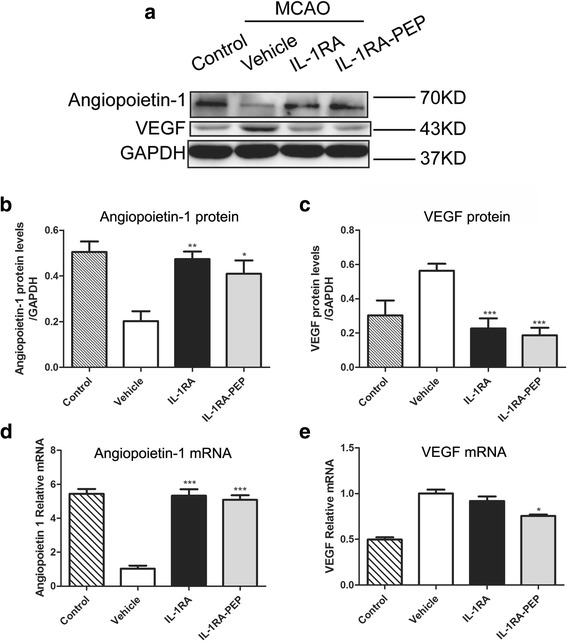
Effects of IL-1RA and IL-1RA-PEP on angiopoietin-1 and VEGF expression in brain tissues following ischemia-reperfusion. **a–c** Protein expression levels of angiopoietin-1 and vascular endothelial growth factor (VEGF) were analyzed by western blot. The gray value of the band was scanned by optical densitometry, and the ratio value was statistically analyzed. **d**, **e** The mRNA expression of *angiopoietin-1* and *VEGF* were analyzed by real-time quantitative PCR. Data are shown as mean ± SEM; *P < 0.05, **P < 0.01, ***P < 0.001 compared to the vehicle group, *n* = 6 per group, based on one-way ANOVA with Bonferroni correction

Blood vessels comprise an essential component of the structure of the BBB. After MCAO induces breakdown of the BBB, angiogenesis occurs [30]. Angiopoietin-1 and VEGF are important factors associated with the process of angiogenesis. Western blot and real-time quantitative PCR analyses were applied to detect the expression of angiopoietin-1 and VEGF in cerebral tissues after transient MCAO. In comparison to the control group, occlusion of the middle cerebral artery remarkably decreased mRNA and protein expression of angiopoietin-1 and notably increased mRNA and protein expression of VEGF. Conversely, in the IL-1RA-PEP treatment group, a marked elevation in angiopoietin-1 expression and reduction in VEGF expression was observed, in comparison to the vehicle group (angiopoietin-1 mRNA, 5.09 ± 0.27 versus 1.03 ± 0.18, *P* < 0.001; angiopoietin-1 protein, 0.41 ± 0.06 versus 0.20 ± 0.04, *P* < 0.05; *VEGF* mRNA, 0.76 ± 0.02 versus 1.00 ± 0.04, P < 0.05; VEGF protein, 0.19 ± 0.04 versus 0.56 ± 0.04, *P* < 0.001). In addition, IL-1RA treatment had a profound influence on the expression of angiopoietin-1 and VEGF (Fig. 6).

### Role of p65/NF-κB pathways on IL-1RA-PEP mediated effects of BBB disruption

**Fig. 7:**
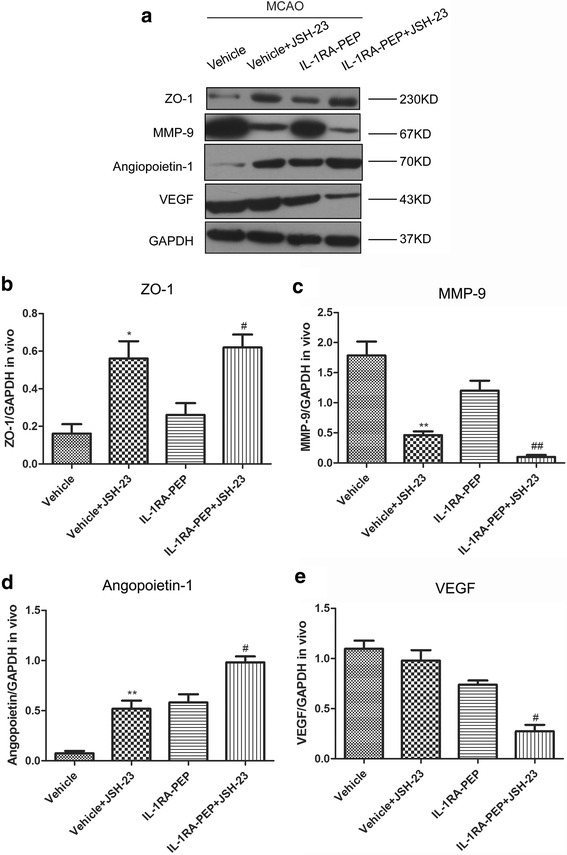
Role of p65/NF-κB pathways in IL-1RA-PEP-mediated effects on blood-brain barrier (BBB) disruption. **a** After the JSH-23 inhibitor was administered, expression levels of ZO-1, MMP-9, angiopoietin-1, and VEGF were analyzed by western blot in the different groups. **b–e** The gray value of the band was scanned by optical densitometry, and the ratio value was statistically analyzed. Data are shown as mean ± SEM; *P < 0.05, **P < 0.01, ***P < 0.001 compared to the vehicle group without JSH-23; #*P* < 0.05, ##*P* < 0.01 compared to the IL-1RA-PEP group without JSH-23; *n* = 4 per group, based on one-way ANOVA with Bonferroni correction

The JSH-23 inhibitor of p65 phosphorylation was used to block the p65/NF-κB pathway. In comparison to the vehicle group, the expression of ZO-1, MMP-9, and angiopoietin-1 in the vehicle + JSH-23 group was significantly altered 24 h after ischemia-reperfusion injury (Fig. 7). The data indicate that the NF-κB pathway significantly regulated the function of the TJ proteins and angiogenic factors.

Moreover, in comparison to the IL-1RA-PEP group, 24 h after transient MCAO, the expression of ZO-1, MMP-9, angiopoietin-1, and VEGF in the IL-1RA-PEP + JSH-23 group was significantly changed (Fig. 7). The data show that after administration of the p65/NF-κB inhibitor, the effects of IL-1RA-PEP on ZO-1, MMP-9, angiopoietin-1, and VEGF were altered.

Therefore, our results indicate that the p65/NF-κB signaling pathway was involved in IL-1RA-PEP-mediated protection against BBB disruption, induced by ischemia-reperfusion injury.

## Discussion

The IL-1RA protein is a naturally occurring antagonist of pro-inflammatory cytokines, including IL-1β and IL-1α. These pro-inflammatory cytokines (IL-1β and IL-1α) are involved in the underlying mechanism of various chronic inflammatory CNS conditions, such as stroke [31], Alzheimer’s disease [32], Parkinson’s disease [33], and epilepsy [34]. Additionally, many studies have confirmed that pro-inflammatory cytokines (IL-1β and IL-1α) are associated with the expression and functions of TJ proteins [35], matrix metalloproteinases [36], and angiogenic factors [37, 38] that provoke the underlying mechanism of BBB disruption. However, there are no studies to prove that IL-1RA can effectively ameliorate the detrimental effects of pro-inflammatory cytokines (IL-1β and IL-1α) on BBB disruption. Thus, in the current study, we defined the potential therapeutic effects of IL-1RA on BBB disruption induced by cerebral ischemia-reperfusion injury, with an aim to restrict the clinical severity and permanent pathology of these injuries.

Despite its outstanding anti-neuroinflammatory effects, certain limitations require attention to make IL-1RA an ideal anti-neuroinflammatory therapeutic agent. These limitations include its relatively high molecular weight, short biological half-life, and low efficiency in brain penetration. To overcome these challenges, we applied the delivery strategy of fusing IL-1RA with a CPP, PEP-1, to construct the bi-functional fusion protein, IL-1RA-PEP. We hope that enhancing brain penetration might lead to improved curative effects of IL-1RA in the treatment of BBB disruption.

To our knowledge, the current study was the first to report the novel finding that intravenous infusions of IL-1RA-PEP had enhanced brain penetration compared to IL-1RA, in rats subjected to MCAO. In addition, the administration of IL-1RA and IL-1RA-PEP reduced disruption of BBB integrity induced by ischemia-reperfusion injury. These findings were confirmed by the EB extravasation, leakage of endogenous IgG, BBB ultrastructure, and permeability of FITC-dextran tracer tests that yielded comparable results among the different treatment groups 24 h after MCAO. The current results also show that IL-1RA and IL-1RA-PEP had regulatory effects on changes in the expression and localization of TJ proteins (ZO-1, occludin, and claudin-5), matrix metalloproteinases (MMP-2 and MMP-9), and angiogenic factors (Ang-1 and VEGF) in the ischemic cerebral tissues of rats.

Studies have proven that the expression of ZO-1, MMPs, and VEGF in different diseases can be regulated by the p65/NF-κB pathway [39–44]. This finding is consistent with the results of the current study, as shown in Fig. 7. Our findings indicate that when the JSH-23 inhibitor was used to block the p65/NF-κb pathway, expression of the TJ proteins and angiogenic factors in ischemia cerebral tissues were different between the vehicle group and the vehicle + JSH-23 group. Furthermore, after the p65/NF-κB pathway was inhibited, the effects of IL-1RA-PEP on the expression of ZO-1, MMP-9, angiopoietin-1, and VEGF were also altered.

Moreover, TJ proteins and angiogenic factors are necessary for BBB formation and stability. Thus, these results when considered altogether indicate that the p65/NF-κB signaling pathway was closely associated with IL-1RA-PEP-mediated protection against BBB disruption induced by ischemia-reperfusion injury. Furthermore, we confirmed in our previous study that IL-1RA-PEP could regulate the phosphorylation of p65 and IκB units in the NF-κB signaling pathway [24]. Therefore, we speculate that the effects of IL-1RA-PEP in preserving BBB integrity might be closely correlated with its regulation of p65/NF-κB pathways. A more in-depth future study would be conducted to elucidate the associated underlying mechanisms.

Related work has shown that the infusion of IL-1β neutralizing antibodies could reduce ischemia-related increases in BBB permeability [45]. These findings are consistent with our results that inhibition of IL-1 function attenuates ischemia-related BBB disruption. However, in Chen et al.’s study [45], the anti-IL-1β antibodies is capability of a selectively neutralizing mAb for IL-1β protein to attenuated BBB dysfunction after ischemia in the ovine fetus. In contrast, in our current study, IL-1RA as a naturally occurring anti-inflammatory cytokine is able to competitively combine the IL-1R to antagonize signal transduction of the IL-1 family (IL-1α and IL-1β), and block the synthesis and action of downstream inflammatory mediators. Therefore, anti-IL-1β mAb and IL-1RA exhibit relatively diverse effects, in terms of their suppression of inflammatory factor (IL-6 and TNF-α) expression and their influence on TJ proteins expression.

In the current study, our findings demonstrated that intravenous infusions of Il-1RA and IL-1RA-PEP effectively attenuated BBB disruption after ischemia-reperfusion injury in male rats. However, females can also be affected by strokes and are the focus of ischemia studies at the NIH in the USA. The existence of physiological differences between female and male subjects could have an influence on the clinically relevant treatment of therapeutic agents. In follow-up studies, it would be of great interest to examine whether Il-1RA and IL-1RA-PEP treatments would also be effective in reducing the ischemia-induced loss of BBB integrity in female rats.

## Conclusion

**Fig. 8:**
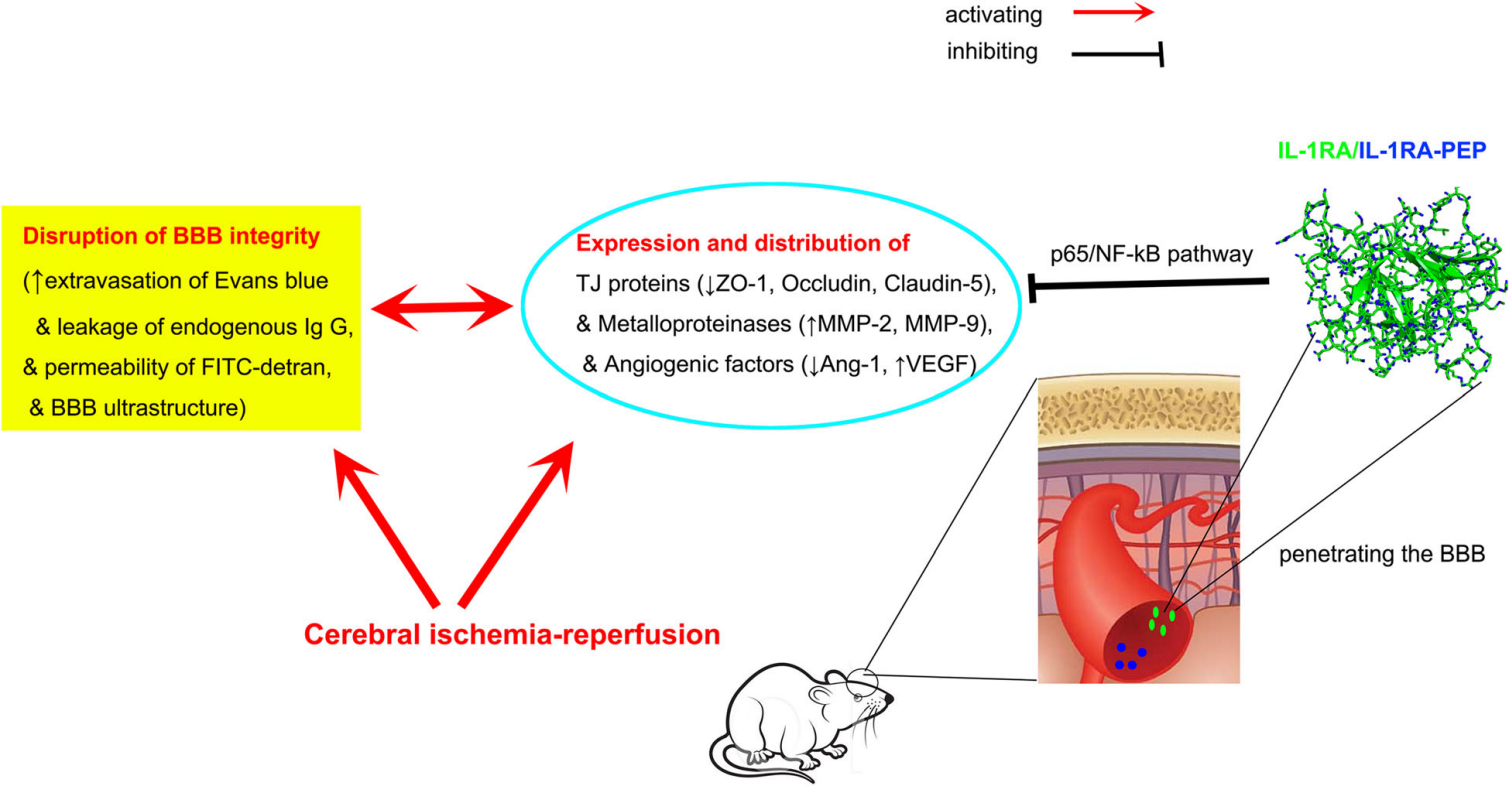
Graphic summary for the effect of IL-1RA and IL-1RA-PEP on BBB disruption in rats subjected to cerebral ischemia-reperfusion injury

After individual intravenous administration, IL-1RA and IL-1RA-PEP penetrated the brains of rats subjected to MCAO, effectively diminished ischemia-induced BBB disruption, and regulated the changes in expression and localization of TJ proteins (Fig. 8). Therefore, systemic IL-1RA and IL-1RA-PEP treatment could be a promising curative approach to ischemic stroke.

## Abbreviations

BBB: Blood-brain barrier
CPPs: Cell-penetrating peptides
DAPI: 4′,6-Diamidino-2-phenylindole
EB: Evans blue
FITC-dextran: Fluorescein isothiocyanate-dextran
GAPDH: Glyceraldehyde 3-phosphate dehydrogenase
ICA: Internal carotid artery
IL-1: Interleukin 1
IL-1R: Interleukin-1 receptor
IL-1RA: Interleukin-1 receptor antagonist
MCAO: Middle cerebral artery occlusion
MMPs: Matrix metalloproteinases
MVECs: Microvascular endothelial cells
PB: Phosphate buffer
PBS: Phosphate-buffered saline
PFA: Paraformaldehyde
RIPA: Radioimmunoprecipitation assay
SAH: Subarachnoid hemorrhage
TEM: Transmission electron microscopy
TJ: Tight junction
VEGF: Vascular endothelial growth factor

## References

[1] Obermeier B, Verma A, Ransohoff RM (2016). The blood-brain barrier. Handb Clin Neurol.

[2] Tajes M, Ramos-Fernandez E, Weng-Jiang X, Bosch-Morato M, Guivernau B, Eraso-Pichot A, Salvador B, Fernandez-Busquets X, Roquer J, Munoz FJ (2014). The blood-brain barrier: structure, function and therapeutic approaches to cross it. Mol Membr Biol.

[3] Haley MJ, Lawrence CB (2017). The blood-brain barrier after stroke: structural studies and the role of transcytotic vesicles. J Cereb Blood Flow Metab.

[4] Gu Y, Dee CM, Shen J (2011). Interaction of free radicals, matrix metalloproteinases and caveolin-1 impacts blood-brain barrier permeability. Front Biosci (Schol Ed).

[5] Khatri R, McKinney AM, Swenson B, Janardhan V (2012). Blood-brain barrier, reperfusion injury, and hemorrhagic transformation in acute ischemic stroke. Neurology.

[6] Pan J, Konstas AA, Bateman B, Ortolano GA, Pile-Spellman J (2007). Reperfusion injury following cerebral ischemia: pathophysiology, MR imaging, and potential therapies. Neuroradiology.

[7] Suzuki Y, Nagai N, Umemura K (2016). A review of the mechanisms of blood-brain barrier permeability by tissue-type plasminogen activator treatment for cerebral ischemia. Front Cell Neurosci.

[8] Rosenberg GA (2012). Neurological diseases in relation to the blood-brain barrier. J Cereb Blood Flow Metab.

[9] Yang Y, Rosenberg GA (2011). Blood-brain barrier breakdown in acute and chronic cerebrovascular disease. Stroke.

[10] Almutairi MM, Gong C, Xu YG, Chang Y, Shi H (2016). Factors controlling permeability of the blood-brain barrier. Cell Mol Life Sci.

[11] Liu WY, Wang ZB, Zhang LC, Wei X, Li L (2012). Tight junction in blood-brain barrier: an overview of structure, regulation, and regulator substances. CNS Neurosci Ther.

[12] Rempe RG, Hartz AM, Bauer B (2016). Matrix metalloproteinases in the brain and blood-brain barrier: versatile breakers and makers. J Cereb Blood Flow Metab.

[13] Engelhardt B, Liebner S (2014). Novel insights into the development and maintenance of the blood-brain barrier. Cell Tissue Res.

[14] Hannum CH, Wilcox CJ, Arend WP, Joslin FG, Dripps DJ, Heimdal PL, Armes LG, Sommer A, Eisenberg SP, Thompson RC (1990). Interleukin-1 receptor antagonist activity of a human interleukin-1 inhibitor. Nature.

[15] Emsley HC, Smith CJ, Georgiou RF, Vail A, Hopkins SJ, Rothwell NJ, Tyrrell PJ (2005). A randomised phase II study of interleukin-1 receptor antagonist in acute stroke patients. J Neurol Neurosurg Psychiatry.

[16] Singh N, Hopkins SJ, Hulme S, Galea JP, Hoadley M, Vail A, Hutchinson PJ, Grainger S, Rothwell NJ, King AT, Tyrrell PJ (2014). The effect of intravenous interleukin-1 receptor antagonist on inflammatory mediators in cerebrospinal fluid after subarachnoid haemorrhage: a phase II randomised controlled trial. J Neuroinflammation.

[17] Helmy A, Guilfoyle MR, Carpenter KL, Pickard JD, Menon DK, Hutchinson PJ (2014). Recombinant human interleukin-1 receptor antagonist in severe traumatic brain injury: a phase II randomized control trial. J Cereb Blood Flow Metab.

[18] Lan KM, Tien LT, Pang Y, Bhatt AJ, Fan LW (2015). IL-1 receptor antagonist attenuates neonatal lipopolysaccharide-induced long-lasting learning impairment and hippocampal injury in adult rats. Toxicol Lett.

[19] Wang F, Wang Y, Zhang X, Zhang W, Guo S, Jin F (2014). Recent progress of cell-penetrating peptides as new carriers for intracellular cargo delivery. J Control Release.

[20] Farkhani SM, Valizadeh A, Karami H, Mohammadi S, Sohrabi N, Badrzadeh F (2014). Cell penetrating peptides: efficient vectors for delivery of nanoparticles, nanocarriers, therapeutic and diagnostic molecules. Peptides.

[21] Zhang D, Wang J, Xu D (2016). Cell-penetrating peptides as noninvasive transmembrane vectors for the development of novel multifunctional drug-delivery systems. J Control Release.

[22] Morris MC, Depollier J, Mery J, Heitz F, Divita G (2001). A peptide carrier for the delivery of biologically active proteins into mammalian cells. Nat Biotechnol.

[23] Longa EZ, Weinstein PR, Carlson S, Cummins R (1989). Reversible middle cerebral artery occlusion without craniectomy in rats. Stroke.

[24] Zhang DD, Zou MJ, Zhang YT, Fu WL, Xu T, Wang JX, Xia WR, Huang ZG, Gan XD, Zhu XM, Xu DG (2017). A novel IL-1RA-PEP fusion protein with enhanced brain penetration ameliorates cerebral ischemia-reperfusion injury by inhibition of oxidative stress and neuroinflammation. Exp Neurol.

[25] Shim KH, Jeong KH, Bae SO, Kang MO, Maeng EH, Choi CS, Kim YR, Hulme J, Lee EK, Kim MK, An SS (2014). Assessment of ZnO and SiO2 nanoparticle permeability through and toxicity to the blood-brain barrier using Evans blue and TEM. Int J Nanomedicine.

[26] Liu M, Shen J, Zou F, Zhao Y, Li B, Fan M (2017). Effect of ulinastatin on the permeability of the blood-brain barrier on rats with global cerebral ischemia/reperfusion injury as assessed by MRI. Biomed Pharmacother.

[27] Leak RK, Zhang L, Stetler RA, Weng Z, Li P, Atkins GB, Gao Y, Chen J (2013). HSP27 protects the blood-brain barrier against ischemia-induced loss of integrity. CNS Neurol Disord Drug Targets.

[28] Jiao H, Wang Z, Liu Y, Wang P, Xue Y (2011). Specific role of tight junction proteins claudin-5, occludin, and ZO-1 of the blood-brain barrier in a focal cerebral ischemic insult. J Mol Neurosci.

[29] Seo JH, Guo S, Lok J, Navaratna D, Whalen MJ, Kim KW, Lo EH (2012). Neurovascular matrix metalloproteinases and the blood-brain barrier. Curr Pharm Des.

[30] Tang Y, Wang L, Wang J, Lin X, Wang Y, Jin K, Yang GY (2016). Ischemia-induced angiogenesis is attenuated in aged rats. Aging Dis.

[31] Denes A, Pinteaux E, Rothwell NJ, Allan SM (2011). Interleukin-1 and stroke: biomarker, harbinger of damage, and therapeutic target. Cerebrovasc Dis.

[32] Shaftel SS, Griffin WS, O'Banion MK (2008). The role of interleukin-1 in neuroinflammation and Alzheimer disease: an evolving perspective. J Neuroinflammation.

[33] Pott Godoy MC, Tarelli R, Ferrari CC, Sarchi MI, Pitossi FJ (2008). Central and systemic IL-1 exacerbates neurodegeneration and motor symptoms in a model of Parkinson’s disease. Brain.

[34] Rijkers K, Majoie HJ, Hoogland G, Kenis G, De Baets M, Vles JS (2009). The role of interleukin-1 in seizures and epilepsy: a critical review. Exp Neurol.

[35] Labus J, Hackel S, Lucka L, Danker K (2014). Interleukin-1beta induces an inflammatory response and the breakdown of the endothelial cell layer in an improved human THBMEC-based in vitro blood-brain barrier model. J Neurosci Methods.

[36] Liang KC, Lee CW, Lin WN, Lin CC, Wu CB, Luo SF, Yang CM (2007). Interleukin-1beta induces MMP-9 expression via p42/p44 MAPK, p38 MAPK, JNK, and nuclear factor-kappaB signaling pathways in human tracheal smooth muscle cells. J Cell Physiol.

[37] Yang L, Guo XG, Du CQ, Yang JX, Jiang DM, Li B, Zhou WJ, Zhang FR (2012). Interleukin-1 beta increases activity of human endothelial progenitor cells: involvement of PI3K-Akt signaling pathway. Inflammation.

[38] Fan F, Stoeltzing O, Liu W, McCarty MF, Jung YD, Reinmuth N, Ellis LM (2004). Interleukin-1beta regulates angiopoietin-1 expression in human endothelial cells. Cancer Res.

[39] Ruhul Amin AR, Senga T, Oo ML, Thant AA, Hamaguchi M (2003). Secretion of matrix metalloproteinase-9 by the proinflammatory cytokine, IL-1beta: a role for the dual signalling pathways, Akt and Erk. Genes Cells.

[40] Kimura K, Teranishi S, Nishida T (2009). Interleukin-1beta-induced disruption of barrier function in cultured human corneal epithelial cells. Invest Ophthalmol Vis Sci.

[41] Chapouly C, Tadesse Argaw A, Horng S, Castro K, Zhang J, Asp L, Loo H, Laitman BM, Mariani JN, Straus Farber R (2015). Astrocytic TYMP and VEGFA drive blood-brain barrier opening in inflammatory central nervous system lesions. Brain.

[42] Huang T, Gao D, Hei Y, Zhang X, Chen X, Fei Z (2016). D-allose protects the blood brain barrier through PPARgamma-mediated anti-inflammatory pathway in the mice model of ischemia reperfusion injury. Brain Res.

[43] Egashira Y, Suzuki Y, Azuma Y, Takagi T, Mishiro K, Sugitani S, Tsuruma K, Shimazawa M, Yoshimura S, Kashimata M (2013). The growth factor progranulin attenuates neuronal injury induced by cerebral ischemia-reperfusion through the suppression of neutrophil recruitment. J Neuroinflammation.

[44] Wu L, Jiang Y, Zhu J, Wen Z, Xu X, Xu X, Xie Y, Yang L, Xu L, Lan W (2014). Orosomucoid1: involved in vascular endothelial growth factor-induced blood-brain barrier leakage after ischemic stroke in mouse. Brain Res Bull.

[45] Chen X, Sadowska GB, Zhang J, Kim JE, Cummings EE, Bodge CA, Lim YP, Makeyev O, Besio WG, Gaitanis J (2015). Neutralizing anti-interleukin-1beta antibodies modulate fetal blood-brain barrier function after ischemia. Neurobiol Dis.

